# Bariatric Surgery: Psychosocial Aspects and Quality of Life

**DOI:** 10.3390/ijerph192416516

**Published:** 2022-12-08

**Authors:** Valentina Martinelli, Matteo Chiappedi

**Affiliations:** 1 Department of Brain and Behavioral Sciences, University of Pavia, via A. Bassi 21, 27100 Pavia, Italy; 2 U.O.S. Neuropsichiatria dell’Infanzia e dell’Adolescenza—Vigevano—ASST Pavia, 27100 Pavia, Italy

Obesity is a major worldwide health problem, causing an ongoing and decades-long pandemic, which the WHO has termed the “global obesity epidemic”, concurrent with the COVID-19 pandemic [1]. The excess of adipose tissue triggers many metabolic and immunological pathways, leading to serious co-morbidities, such as impaired glucose tolerance (type 2 diabetes in the worst cases), dyslipidemia, arterial hypertension, hyperuricemia, obstructive sleep apnea, polycystic ovary syndrome, or non-alcoholic fatty liver disease.

Although lifestyle modifications and pharmacological interventions are often exploited as first-line interventions, it is clear that bariatric surgery is the most effective strategy, especially in more severe forms of obesity [2]. It has been reported, however, that results are improved if a multi-disciplinary management is activated, in line with the multiple aspects involved in obesity [3,4]. According to the American Society for Gastrointestinal Endoscopy (ASGE), a multidisciplinary team should include an endocrinologist and/or obesity medicine physician, a bariatric surgeon, an endoscopist experienced in bariatrics, an anaesthesiologist, a registered dietitian, an exercise specialist, a behaviour coach, a psychologist, and a nurse or physician extender that coordinates the team [5].

A number of aspects regarding bariatric surgery are still debated. Some remaining open questions are directly connected to surgical procedures. Firstly, although laparoscopic sleeve gastrectomy is considered the current technique of choice, given its efficacy and the reduced impact of side effects compared to other interventions [3], a number of other options are available and could be better suited in specific situations, including “traditional” Roux-en-Y-gastric by-pass, vertical-banded gastroplasty, and laparoscopic Roux-en-Y-gastric by-pass.

Another relevant aspect that has only recently been studied in detail is the metabolic effect of bariatric surgery. Moreover, a recent paper suggested that to manage the complexity of metabolomics it could be useful to exploit artificial intelligence [6]. However, it is clear that any form of bariatric surgery has a significant impact on nutrients absorption but also on other aspects, including changes in food choice, taste, desire and enjoyment [7].

The results of bariatric surgery were initially evaluated in terms of the reduction in body weight or body mass index. Data concerning psychosocial effects are interesting and seem to confirm an improvement in the Quality of Life of bariatric patients by increasing their possibility of having a complete social life [8]. The additional utility of psychological support in improving psychosocial outcomes of bariatric surgery, compared to a merely surgical and medical approach, is less clear: a recent review of research, applying a Bayesian approach, found very uncertain evidence and suggested more high quality studies in the field [9]. However, it is clear that bariatric surgery requires a multi-disciplinary approach to manage patients’ difficulties in achieving healthier eating [2], and this kind of intervention can also be useful to improve self-reported mental health and quality of life, in addition to reducing cardiovascular risk factors [10]. In this context, it is not surprising that few studies attempted to link specific pre-operative factors to psychosocial results and/or to the need for psychological or psychiatric intervention. However, this line of research is promising, as evidenced by a recent study in candidates of bariatric surgery where a reduced score in the mental domain of Quality of Life, as explored by the 12-item Short Form Health Survey, was associated with a lower pre-treatment BMI [11], with a possible impact on surgical outcomes. Moreover, epidemiological data and clinical research suggest that bariatric surgery may hamper some patients’ mental health, with an increased risk of self-harm and suicide [12].

Almost all the above-mentioned studies were conducted on adult subjects. Only in recent times has a growing body of literature concerning adolescents been published. It is clear that bariatric surgery, and especially laparoscopic sleeve gastrectomy, is at least as effective as in adults [13]; on the other hand, the possibility of obtaining results in terms of quality of life and psychosocial adjustment has been studied in far less detail.

The main objective of this Special Issue is to provide cutting-edge data regarding psychosocial aspects of bariatric surgery, including treatment options and ethical implications across different age groups.
